# Economic Evaluation of Thymectomy for the Treatment of Nonthymomatous Myasthenia Gravis

**DOI:** 10.1001/jamanetworkopen.2026.6612

**Published:** 2026-04-13

**Authors:** Joseph Lord, Matthew Walton, Peter Murphy, Lucy Shepherd, Gil I. Wolfe, Jon Sussman, Gary Cutter, Henry Kaminski, Inmaculada Aban, Ali G. Hamedani, Robert Hodgson

**Affiliations:** 1 Sheffield Centre for Health and Related Research (SCHARR), University of Sheffield, Sheffield, United Kingdom; 2 Centre for Reviews and Dissemination (CRD), University of York, Alcuin College, York, United Kingdom; 3 LSE Health, London School of Economics and Political Science, London, United Kingdom; 4 Department of Neurology, University at Buffalo Jacobs School of Medicine and Biomedical Sciences/SUNY, Buffalo, New York; 5 Department of Neurology, Greater Manchester Neuroscience Centre, Salford, Greater Manchester, United Kingdom; 6 Department of Biostatistics, University of Alabama at Birmingham; 7 Department of Neurology & Rehabilitation Medicine, George Washington University School of Medicine and Health Sciences, Washington, DC; 8 Department of Neurology, University of Pennsylvania, Philadelphia

## Abstract

**Importance:**

Myasthenia gravis (MG) is a chronic autoimmune disease that significantly impairs patients’ quality of life by causing fluctuating muscle weakness and fatigue. While pharmacological treatment with corticosteroids remains a cornerstone of treatment, long-term use is often associated with substantial adverse effects. Although thymectomy is an established and effective alternative and adjunct to pharmacological treatment, its long-term cost-effectiveness within the UK National Health Service (NHS) is unknown.

**Objective:**

To evaluate the cost-effectiveness of thymectomy in addition to prednisolone therapy compared with prednisolone therapy alone for patients with nonthymomatous MG.

**Design, Setting, and Participants:**

This economic evaluation used a Markov state-transition model to simulate the costs and health outcomes of patients with nonthymomatous MG. The patients’ baseline characteristics, disease progression, and treatment efficacy were derived from patient-level data from the Thymectomy Trial in Nonthymomatous Myasthenia Gravis Patients Receiving Prednisone Therapy (MGTX) clinical trial. The model adopted a UK NHS and Personal Social Services perspective over a lifetime horizon. Data analysis was performed from June 2024 to October 2025.

**Main Outcomes and Measures:**

The primary outcomes were incremental costs and quality-adjusted life years (QALYs) discounted at 3.5% per annum and the incremental cost-effectiveness ratio (ICER). Extensive deterministic and probabilistic sensitivity analyses were conducted to assess the robustness of the findings to parameter uncertainty.

**Results:**

There were 126 participants in the MGTX trial, with a mean age of 35.08 years; 70.63% were female. In the base-case analysis, thymectomy plus prednisolone dominated prednisolone alone, yielding higher health gains (14.63 QALYs vs 14.11 QALYs) and lower lifetime costs (£181 716 vs £194 730). This generated an incremental gain of 0.52 QALYs and a cost saving of £13 014 per patient. The results were robust across a wide range of sensitivity analyses. Probabilistic analysis (1000 simulations) showed that thymectomy had a greater than 99% probability of being cost-effective at a willingness-to-pay threshold of £25 000 per QALY.

**Conclusions and Relevance:**

In this economic evaluation, thymectomy was found to be a highly cost-effective intervention for nonthymomatous MG in the UK and likely to be cost-saving relative to prednisolone alone. These findings support current clinical guidelines recommending thymectomy for eligible patients and establish it as the most relevant comparator in evaluations of future health technologies. More broadly, this study demonstrates the importance of high-quality randomized clinical trial evidence in enabling health technology assessment of surgical procedures.

## Key Points

**Question:**

Is thymectomy a cost-effective treatment option for nonthymomatous myasthenia gravis (MG) in the UK National Health Service (NHS)?

**Findings:**

In this model-based economic evaluation, thymectomy plus prednisolone resulted in a lifetime incremental quality-adjusted life year gain of 0.52 and a cost saving of £13 014 per patient, dominating a strategy of prednisolone alone.

**Meaning:**

The findings of this study suggest that thymectomy is a highly cost-effective treatment option for patients with nonthymomatous MG in the UK NHS.

## Teaser

This economic evaluation compares the cost-effectiveness of thymectomy as a treatment for patients with nonthymomatous myasthenia gravis from the perspective of the UK National Health Service.

## Introduction

Myasthenia gravis (MG) is a chronic autoimmune disease affecting the neuromuscular junction, characterized by antibodies to postsynaptic proteins, most commonly to the nicotinic acetylcholine receptor (AChR).^1^ The resulting neuromuscular dysfunction leads to weakness and fatigue of skeletal muscles, typically affecting ocular, bulbar, and proximal limb skeletal muscle groups.^2,3^ Ocular symptoms, namely diplopia and ptosis, are a common initial clinical presentation, with approximately 80% of patients progressing to more generalized weakness.^4^

The burden of disease for patients with MG is substantial, profoundly affecting physical functioning and mental health. Impaired mobility and reduced endurance limit patients’ ability to perform daily activities, while the unpredictable nature of symptoms can contribute to anxiety, depression, and social withdrawal, diminishing a patient’s sense of independence and overall quality of life.^5,6,7^

Currently, there is no cure for MG. Pharmacological management aims to achieve symptom control, maintain remission or minimal manifestation status, and prevent myasthenic exacerbations and crises. UK guidelines recommend initiating treatment with the acetylcholinesterase inhibitor, pyridostigmine, at the lowest effective dose,^8^ which is sufficient to achieve symptom control in some patients with mild disease.^8,9^ However, most patients require immunosuppression with corticosteroids, such as prednisolone (in the UK) or prednisone (in the US), often alongside other immunosuppressants such as azathioprine. While effective, long-term corticosteroid use is associated with adverse events, including weight gain, cataracts, osteoporosis, metabolic disorders, and less commonly, peptic ulcers and serious infections.^10,11,12^ Steroid-sparing treatment strategies are thus a key clinical aim.

Surgical management using thymectomy is an important adjunct to pharmacological treatment for patients with generalized, AChR-seropositive MG who are younger than 45 years.^8^ Historically, early thymectomy was believed to reduce long-term steroid use requirements and increase the likelihood of achieving remission. However, these assumptions were largely based on observational or case series data and lacked rigorous clinical evaluation.^13,14,15^ The Thymectomy Trial in Nonthymomatous Myasthenia Gravis Patients Receiving Prednisone Therapy (MGTX) randomized clinical trial (RCT) compared extended open transsternal thymectomy plus prednisone vs prednisone alone in nonthymomatous MG.^16,17^ Over a 5-year period, thymectomy improved the time-weighted average quantitative MG (QMG) scores, reduced prednisone requirements, and resulted in fewer hospitalizations for myasthenic exacerbation, resolving long-standing uncertainty regarding its clinical benefit.^16,17^

While the effectiveness of thymectomy is now well supported, there is no evidence supporting the cost-effectiveness of this procedure in a UK context. To our knowledge, no previous studies have assessed the economic value of thymectomy for patients with nonthymomatous MG.^18,19^ Thymectomy is associated with substantive up-front procedural costs; therefore, it is uncertain whether the long-term clinical benefits of thymectomy justify these costs. This study evaluates whether thymectomy plus prednisolone is a cost-effective treatment for nonthymomatous MG in the UK National Health Service (NHS).

## Methods

Per University Research Governance policy, ethical approval was not required for this study as the economic evaluation was based on the secondary use of deidentified data or data in the public domain. For this economic evaluation, a Markov decision-analytic model was developed to estimate the long-term costs and health outcomes of thymectomy for the treatment of nonthymomatous MG compared with pharmacological care (considered to be the range of medications and treatments received by patients on the care pathway in the UK NHS). The model was conceptualized according to best practice guidelines^20^ with guidance from a project advisory group of UK and US clinical experts. No formal protocol was published. Model inputs were primarily informed by data from the MGTX trial,^16^ supplemented with data obtained from published literature. The model was built in Excel version 2512 (Microsoft Corp) using Visual Basic for Applications. Data analysis was performed from June 2024 to October 2025.

The economic analysis adopted a UK NHS and Personal Social Services perspective, and therefore, only direct costs were considered. Costs and outcomes were discounted at 3.5% per annum,^21^ and the base case estimated outcomes from a lifetime time horizon (65 years). This study followed the Consolidated Health Economic Evaluation Reporting Standards (CHEERS) guideline.^22^

### Model Population

The baseline characteristics of the model population were derived from patients enrolled into the MGTX trial (N = 126). The model population therefore reflects patients with early-stage generalized MG, and results should be interpreted in this clinical context. The cohort population had a mean age of 35.08 years, and 70.63% were female, comparable with UK thymectomy populations.^23^

### Model Structure

**Figure 1.**
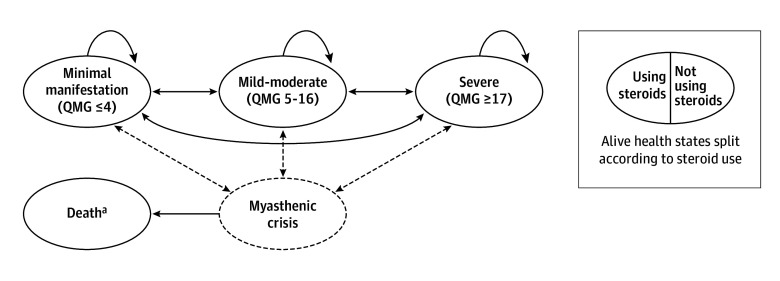
Graphical Markov Model Structure ^a^Death state is absorbing; natural mortality possible from any state.

The model structure is shown in Figure 1 and comprises 3 mutually exclusive alive health states, with health state membership defined according to QMG score, a standardized clinical tool used to assess the severity of muscle weakness in patients with MG. Health states were classified using the following QMG thresholds: minimal manifestation (QMG ≤4), mild to moderate (QMG 5-16), and severe (QMG ≥17). Thresholds were informed by published cutoffs^24,25,26^ and validated by clinical experts; it is noted that these QMG ranges do not represent a precise clinical classification. Patients enter the model according to the baseline severity distribution observed in MGTX: minimal manifestation (5.7%), mild to moderate (73.4%), and severe (20.5%).

In each 4-week model cycle, patients can remain in their current state, move to a more or less severe health state, experience myasthenic crisis, or transition to the absorbing death state. A tunnel state is implemented to capture the acute costs and health consequences of myasthenic crisis, where transition is possible from any alive state at the start of a given cycle. At the end of the cycle, the patient can either return to their original health state or experience mortality. Myasthenic exacerbation was tracked in the model but was not associated with an increased mortality risk.

### Complications of Long-Term Corticosteroid Use

Due to limited published evidence on the health economic impact of long-term corticosteroid use, this was not included in the base case. An exploratory scenario adopted the approach described in the National Institute for Health and Care Excellence (NICE) appraisal of efgartigimod.^27^ In this scenario, a per-cycle cost, utility decrement, and standardized mortality ratio (SMR) are applied to patients taking corticosteroids in the model, stratified by those on a high dose and those receiving a low dose.

### Mortality

In the base case, patients were subject to age- and sex-matched general population mortality rates, an assumption based on clinical advice and a lack of data supporting an increased risk of mortality associated with MG beyond that of the general population. During myasthenic crisis, an additional fixed mortality probability of 4.47% was applied, based on a large study of hospitalized MG patients in the US.^28^

### Myasthenic Crisis and Exacerbation

The model explicitly represents myasthenic crisis as a tunnel state where patients in any health state are at risk according to state-specific probabilities derived from MGTX (Table 1).^16,27,28,29,30,31,32,33,34,35^ Myasthenic exacerbation is implemented in a similar way with state-specific probabilities (also derived from MGTX) determining the frequency of exacerbation. Both crisis and exacerbation incur a utility decrement and a cost; however, unlike crisis, exacerbation is not associated with an increased risk of mortality. For the purpose of calculating utilities, myasthenic crisis was assumed to last for 31.1 days and exacerbation 11.8 days.^34^

**Table 1.** Model Parameters

Parameter	Value	Standard error	Distribution for PSA	Source
Surgery costs, £				
Thymectomy	9730.00	20% of Mean	Gamma	Personal communication^a^
Drug costs per 100 mg, £				
Prednisolone	0.53	20% of Mean	Gamma	NICE^29^
Pyridostigmine	0.24	20% of Mean	Gamma	NICE^29^
Azathioprine	0.04	20% of Mean	Gamma	NICE^29^; Department of Health and Social Care^30^
Cyclosporine	1.71	20% of Mean	Gamma	NICE^29^
Patient monitoring costs, £				
10-min GP appointment	45.00	20% of Mean	Gamma	Jones et al,^31^ 2024
Hospital outpatient	230.00	20% of Mean	Gamma	Jones et al,^31^ 2024
Nurse, hospital	195.76	20% of Mean	Gamma	NHS England^32^
Physiotherapist	93.75	20% of Mean	Gamma	NHS England^33^
Neurologist	232.30	20% of Mean	Gamma	NHS England^33^
Language or speech therapist	100.00	20% of Mean	Gamma	Jones et al,^31^ 2024
1-h Nurse visit	45.00	20% of Mean	Gamma	Jones et al,^31^ 2024
End-of-life costs, £	13 409.00	20% of Mean	Gamma	Jones et al,^31^ 2024
Costs associated with rescue treatments, £				
Myasthenic crisis	36 174.92	£3617.49	Gamma	NICE,^27^ 2025
Myasthenic exacerbation	16 595.23	£1659.52	Gamma	NICE,^27^ 2025
Mortality due to crisis	0.0447	NA	Fixed	Alshekhlee et al,^28^ 2009
Utilities, mean				
Minimal manifestation (QMG score 0-4)	0.7958	0.010	Beta	Wolfe et al,^16^ 2016
Mild to moderate (QMG score 5-16)	0.6711	0.008	Beta	Wolfe et al,^16^ 2016
Severe (QMG score >16)	0.5497	0.023	Beta	Wolfe et al,^16^ 2016
Disutility for myasthenic exacerbation	−0.2000	20% of Mean	Beta	Canada’s Drug Agency, ^34^ 2020
Disutility for myasthenic crisis	−0.7200	20% of Mean	Beta	Canada’s Drug Agency, ^34^ 2020
Duration of exacerbation disutility	11.8 d	NA	Fixed	Canada’s Drug Agency, ^34^ 2020
Duration of crisis disutility	31.1 d	NA	Fixed	Neumann et al,^35^ 2020
Annual event rates				
Crisis				
Minimal manifestation	0.0112	0.0079	Gamma	Wolfe et al,^16^ 2016
Mild to moderate	0.0697	0.0164	Gamma	Wolfe et al,^16^ 2016
Severe	0.3371	0.1066	Gamma	Wolfe et al,^16^ 2016
Exacerbation				
Minimal manifestation	0.1120	0.0250	Gamma	Wolfe et al,^16^ 2016
Mild to moderate	0.2786	0.0328	Gamma	Wolfe et al,^16^ 2016
Severe	0.8764	0.1719	Gamma	Wolfe et al,^16^ 2016
Corticosteroid complications				
HRs				
Low dose	1.11	NA	Fixed	NICE,^27^ 2025
High dose	2.10	NA	Fixed	NICE,^27^ 2025
Utility decrement				
Low dose	0.07	20% of Mean	Beta	NICE,^27^ 2025
High dose	0.18	20% of Mean	Beta	NICE,^27^ 2025
Cycle complication costs, £ per cycle				
Low dose	26.04	20% of Mean	Gamma	NICE,^27^ 2025
High dose	183.34	20% of Mean	Gamma	NICE,^27^ 2025

Abbreviations: GP, general practitioner; HR, hazard ratio; NA, not applicable; NHS, National Health Service; PSA, probabilistic sensitivity analysis; QMG, quantitative myasthenia gravis.

^a^ Information provided by Dionisios Stavroulias, MD, and Chris Middlemass, Oxford University Hospitals NHS Foundation Trust, written communication, December 10, 2024.

### Costs and Resource Use

Cost and resource use reported in the model includes the cost of the procedure itself, the cost of concomitant medications, patient monitoring costs, costs associated with managing myasthenic events, and costs of end-of-life care. State-specific resource use associated with patient monitoring was derived from the NICE appraisal of efgartigimod.^27^ Dosing of prednisolone, pyridostigmine, azathioprine, and cyclosporine were likewise calculated on a state- and treatment group–specific basis, informed by resource use data from the MGTX trial. Historical costs were inflated to 2025 values using the Bank of England inflation calculator.^36^

The cost of the thymectomy procedure was obtained from Oxford University Hospitals NHS Foundation Trust data (Dionisios Stavroulias, MD, and Chris Middlemass, Oxford University Hospitals NHS Foundation Trust, written communication, December 10, 2024). It was estimated as the average cost of video-assisted thoracoscopic surgery (VATS) thymectomy in 2023 to 2024—estimated to be £9730 (to convert to US dollars, multiply by 1.34). It is acknowledged that the cost associated with a transsternal approach (that taken in the MGTX trial) is likely to be higher,^37,38^ but no specific data were available for the UK. As a result, a scenario was explored with a 50% higher procedure cost. An additional scenario analysis also explored the impact of using the procedure cost described in NHS reference costs^39^—a value of £7791 (NHS healthcare resource group code DZ63A: average across Charlson Comorbidity Index score).

### Uncertainty

Parameter uncertainty was explored deterministically by varying input parameters according to plausible ranges. PSA was also conducted where parameter values were sampled from their respective probability distributions (Table 2).^40,41^ Coherent transition probability matrices were sampled from the parametric bootstrap simulations of the multistate model as described later. Where estimates of variance were not available, standard error values were conservatively assumed to be 20% of the corresponding mean value.

**Table 2.** Cycle Transition Probabilities^a^

Starting health state^b^	Transition probabilities by severity of destination health state, mean (SD)					
	Minimal manifestation		Mild to moderate		Severe	
	PA	TPP	PA	TPP	PA	TPP
**Phase 1 (≤1 y)**						
Minimal manifestation	0.8186 (0.0455)	0.9734 (0.0109)	0.1737 (0.0450)	0.0262 (0.0107)	0.0077 (0.0077)	0.0005 (0.0003)
Mild to moderate	0.0927 (0.0175)	0.0575 (0.0110)	0.8832 (0.0191)	0.9127 (0.0151)	0.0241 (0.0085)	0.0298 (0.0112)
Severe	0.0135 (0.0084)	0.0194 (0.0125)	0.1782 (0.0443)	0.4450 (0.1034)	0.8084 (0.0462)	0.5356 (0.1075)
**Phase 2 (>1 y)**						
Minimal manifestation	0.9298 (0.0115)	0.9611 (0.0058)	0.0694 (0.0114)	0.0342 (0.0059)	0.0008 (0.0002)	0.0047 (0.0031)
Mild to moderate	0.0366 (0.0058)	0.0581 (0.0083)	0.9431 (0.0075)	0.9355 (0.0089)	0.0203 (0.0048)	0.0063 (0.0042)
Severe	0.0040 (0.0042)	0.0880 (0.0977)	0.1645 (0.0342)	0.4091 (0.1669)	0.8315 (0.0345)	0.5029 (0.1889)

Abbreviations: PA, prednisolone alone; TPP, thymectomy plus prednisolone.

^a^ Data source was Thymectomy Trial in Nonthymomatous Myasthenia Gravis Patients Receiving Prednisone Therapy trial data.^16^

^b^ Probabilities are for transitioning from the state listed in the row headers (starting state) to the state listed in the column headers (destination state) for any given cycle, dependent on current model phase and treatment arm. For example, the mean (SD) probability of transitioning from minimal manifestation for a cycle in phase 1 to the mild to moderate state the following cycle after having received PA is 0.1727 (0.0450).

### Statistical Analysis

#### Efficacy Parameters and Transition Probabilities

Individual participant data (IPD) from the MGTX trial were used to calculate the probability of transitioning between health states. To reflect the time-dependent nature of the treatment effect observed in the trial, separate transition matrices were calculated for the short-term (≤1 year) and long-term (>1 year) using the multistate modeling package msm in R version 4.5.2 (R Project for Statistical Computing).^42^ Parameter uncertainty in transition probabilities was incorporated using parametric simulation from the fitted multistate models. In each of 2000 bootstrap simulations, a new set of transition parameters was sampled from their estimated sampling distribution and used to construct a full transition probability matrix. These matrices were sampled from directly within the probabilistic sensitivity analysis (PSA), ensuring that each iteration used a coherent set of transition probabilities derived from the underlying trial data.

The transition probability matrices applied in the model are presented in Table 2. The standard deviations reported for transition probabilities in this table represent the empirical variability across bootstrap simulations, rather than distributional parameters used to generate independent probabilistic samples.

#### Long-Term Efficacy

The MGTX RCT and its extension study provide data for up to 5 years of follow-up after thymectomy.^17^ The base-case analysis extrapolates the benefit of thymectomy for 10 years, after which a structural loss of response occurs, such that transition probabilities for the thymectomy group revert to those associated with long-term prednisolone alone for the remainder of the model horizon.

Given uncertainty regarding the true persistence of the treatment effect, we performed threshold analysis to explore the impact of varying the treatment maintenance period (eFigure 2 in Supplement 1). A durable response scenario was also explored, in which the treatment effect was assumed to persist for the full model time horizon.

#### Health State Utilities

All patients in the MGTX study completed the Medical Outcomes Study 36-Item Short Form Health Survey (SF-36)^43^ at regular intervals throughout their participation in the study. These data were collected alongside QMG scores, allowing the assessment of the association between MG severity and health-related quality of life (HRQoL) outcomes. A mapping algorithm published by Kharroubi and colleagues^44^ was used to convert raw SF-36 scores to EQ-5D utilities using UK preference weights.^45^

Linear mixed-effects models were fitted using the lme4 package^46^ in R to estimate the association between significant determinants and EQ-5D scores, accounting for repeated measures within subjects. A total of 567 observations contributed to the final health state utility estimates, which were based on regression coefficients for age and QMG score categories of 0 to 4, 5 to 16, and greater than 16. Each of the covariates included in the final model demonstrated a high level of statistical significance (*P* < .001). The association of aging with the HRQoL of the modeled cohort was incorporated according to UK population norms.^47^ Disutilities associated with myasthenic crisis and exacerbation events were sourced from published literature (Table 1).

## Results

### Main Results

The results of the deterministic base-case analysis are presented in Table 3. In the base-case analysis, assuming a loss of response after 10 years, thymectomy with pharmacological therapy generated an additional 0.52 QALYs and a cost savings of £13 014 per patient compared with pharmacological therapy alone. Thymectomy with pharmacological care therefore dominated pharmacological care alone, being both more effective and less costly. When a durable treatment effect was assumed for the lifetime of the cohort, the benefits of thymectomy were even more pronounced, yielding both greater incremental health benefits (a gain of 1.24 QALYs) and increased cost savings of £49 629.

**Table 3.** Deterministic Results

Long-term efficacy assumption and strategy	Incremental CE							
	Costs, £	QALYs	Incremental costs, £	Incremental QALYs	ICER	iNMB, by CE threshold, £^a^		
						£25 000	£35 000
**Main results**							
Base case							
Loss of response after 10 y							
Thymectomy	181 716	14.63	−13 014	0.52	Strictly dominant	26 104	31 339
Standard care	194 730	14.11					
Durable treatment effect							
Thymectomy	145 101	15.36	−49 629	1.25	Strictly dominant	80 774	93 232
Standard care	194 730	14.11					
Exploratory corticosteroid scenario							
Loss of response after 10 y							
Thymectomy	199 972	12.17	−21 180	1.51	Strictly dominant	58 851	73 919
Standard care	221 152	10.66					
Durable treatment effect							
Thymectomy	163 772	13.14	−57 380	2.48	Strictly dominant	119 365	144 160
Standard care	221 152	10.66					
**Additional scenario analyses**							
Alternative crisis utility decrement (−0.39 for 31.1 d)^b^							
Loss of response after 10 y							
Thymectomy	181 716	14.67	−13 014	0.52	Strictly dominant	25 921	31 084
Standard care	194 730	14.15					
Durable treatment effect							
Thymectomy	145 101	15.38	−49 629	1.23	Strictly dominant	80 300	92 569
Standard care	194 730	14.15					
Alternative exacerbation utility decrement (−0.16 for 20.73 d)^c^							
Loss of response after 10 y							
Thymectomy	181 716	14.62	−13 014	0.53	Strictly dominant	26 144	31 396
Standard care	194 730	14.10					
Durable treatment effect							
Thymectomy	145 101	15.35	−49 629	1.25	Strictly dominant	80 880	93 381
Standard care	194 730	14.10					
Alternative event costs (50% of exacerbation and crisis costs)							
Loss of response after 10 y							
Thymectomy	116 190	14.63	−3210	0.52	Strictly dominant	16 299	21 535
Standard care	119 399	14.11					
Durable treatment effect							
Thymectomy	95 436	15.36	−23 964	1.25	Strictly dominant	55 108	67 566
Standard care	119 399	14.11					
Procedure cost according to NHS reference cost (£7791)							
Loss of response after 10 y							
Thymectomy	179 777	14.63	−14 953	0.52	Strictly dominant	28 043	33 278
Standard care	194 730	14.11					
Durable treatment effect							
Thymectomy	143 162	15.36	−51 568	1.25	Strictly dominant	82 713	95 171
Standard care	194 730	14.11					
Procedure cost 50% higher (£14 595)							
Loss of response after 10 y							
Thymectomy	186 581	14.63	−8149	0.52	Strictly dominant	21 239	26 474
Standard care	194 730	14.11					
Durable treatment effect							
Thymectomy	149 966	15.36	−44 764	1.25	Strictly dominant	75 909	88 367
Standard care	194 730	14.11					

Abbreviations: CE, cost-effectiveness; ICER, incremental cost-effectiveness ratio; iNMB, incremental net monetary benefit; NHS, National Health Service; QALY, quality-adjusted life-year.

^a^ iNMB describes the monetary value associated with an intervention compared with a comparator. A positive iNMB indicates that the intervention is cost-effective at a given cost-effectiveness threshold, calculated as follows: iNMB = (incremental QALYs × threshold) − incremental cost.

^b^ Data source for the alternative crisis utility decrement was Canada’s Drug Agency,^34^ 2020.

^c^ Data source for the alternative exacerbation utility decrement was NICE,^27^ 2025.

### PSA

**Figure 2.**
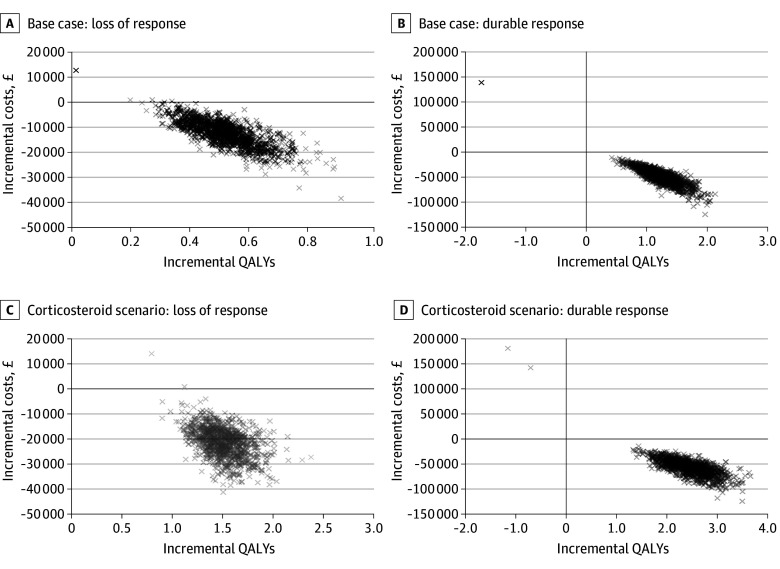
Scatterplots of Probabilistic Sensitivity Analysis Cost-Effectiveness Plane Across all scenarios, more than 99% of the simulations fell in the lower right quadrant, indicating that thymectomy was dominant (ie, more effective and less costly). At a cost-effectiveness threshold of £25,000, more than 99% of simulations were considered cost-effective. QALY indicates quality-adjusted life-year. The output of each 1000 model simulations are marked with an x.

The results of the PSA are shown in the cost-effectiveness planes in Figure 2. Thymectomy was dominant in more than 99% of simulations (ie, more effective and less costly).

### Scenario Analysis

Table 3 presents the results of additional scenario analyses. Scenarios A and B explored different assumptions regarding the utility decrement (and duration) associated with crisis and exacerbation from alternative data sources.^27,34^ Scenario C explored reducing the costs associated with crisis and exacerbation by 50% (no alternative data sources were available). Scenario D explored using the NHS reference cost (DZ63A) to represent the thymectomy procedure cost, and Scenario E explored increasing the procedure cost by 50% to represent the potential additional cost of a transsternal approach. Threshold analysis found that thymectomy remained dominant even if the treatment effect was not modeled to persist beyond the 5-year trial period. An additional analysis reports model-projected events and state occupancy data (eTable in Supplement 1).

### Deterministic Sensitivity Analysis

The impact of varying model inputs according to their plausible minimum and maximum values is shown in eFigure 1 in Supplement 1. The most influential parameters on cost-effectiveness included the cost of the thymectomy procedure itself, the cost attached to myasthenic crisis, mortality risk attached to myasthenic crisis, and the cost of managing myasthenic exacerbation. Despite this, thymectomy remained cost-effective across the plausible ranges of all parameters. Other parameters had little impact on model results.

## Discussion

To our knowledge, this study provides the first UK-based cost-effectiveness analysis of thymectomy in patients with nonthymomatous MG, informed directly by MGTX efficacy and HRQoL IPD. Our analysis found that the significant up-front cost of thymectomy is outweighed by the long-term clinical benefits and subsequent cost offsets, making thymectomy a highly cost-effective option compared with pharmacological management alone. Decision uncertainty was minimal, with a greater than 99% probability of cost-effectiveness in the PSA.

The model reflects a comparison of current NHS management strategies for MG. The procedure costs used in the model (inclusive of hospital stay) reflect VATS thymectomy and therefore align with contemporary minimally invasive practice; most patients are expected to undergo VATS in current NHS care, with outcomes considered comparable with open approaches when performed by adequately trained surgeons.^19,48,49^ At present, newer biologic immunotherapies are not available in routine commissioning within the NHS, and, where assessed by NICE, have been positioned as add-ons to standard care. Thymectomy in early-stage disease is therefore unlikely to displace these treatments if they were recommended, but it could potentially reduce the need for escalation in some patients.

Health gains were driven by sustained improvements in QMG score, with more patients in the thymectomy group achieving and maintaining minimal manifestation status. This translated directly into higher utility values and incremental QALYs, alongside a reduction in the frequency of costly myasthenic exacerbation and crisis events, generating downstream cost savings.

A key driver of the results was the use of IPD to estimate separate short- and long-term transition matrices, allowing risks to vary over time rather than assuming constant hazards. The trial data demonstrated a pronounced early reduction in progression to severe disease, with differential risks persisting beyond the first year and no evidence of hazard convergence between treatments within observed follow-up. Modeling approaches that assume constant or proportionally evolving transition risks may therefore fail to capture the relative economic impacts of the comparator strategies, whose effects vary in magnitude and pattern over time. Utilities were estimated from the same patient population using mixed-effects regression analysis, ensuring internal consistency between clinical progression and quality of life. Together, these findings support the clinical and economic relevance of thymectomy as a benchmark against which emerging advanced therapies should be evaluated in future NICE appraisals.

Our findings remained robust across all scenarios explored, with thymectomy remaining either dominant, or highly cost-effective, considering conventional UK willingness-to-pay thresholds of £25 000 to £35 000 per QALY gained. Even assuming loss of benefit after 5 years, ie, the observed duration of follow-up in MGTX, thymectomy remained dominant. Incorporating the health and cost impacts of long-term corticosteroid-related complications further increased the estimated value of thymectomy.

More broadly, the analysis highlights the importance of generating high-quality RCT evidence on the clinical efficacy of surgical procedures relative to pharmacological management. The MGTX study provides a clear example of how such evidence can inform more comprehensive health technology assessment by bodies such as NICE, ensuring that procedures are appropriately considered and recommended for use through comparisons against pharmaceutical alternatives. Without suitable data for economic modeling, highly cost-effective procedures risk being overlooked in commissioning decisions, with implications for allocative efficiency and patient outcomes.

### Limitations

There are several limitations to our analysis. Although the model was grounded in high-quality data from MGTX, this trial was not conducted exclusively in the UK,^50^ and health care utilization patterns may differ from those in the NHS; UK-specific cost data and clinical input were used to mitigate this. Long-term data on the durability of thymectomy’s outcomes beyond 5 years are limited, and the 10-year treatment effect assumption is a pragmatic choice informed by the observed stability of hazards within trial follow-up. While the results were insensitive to this assumption, longer-term observational data would strengthen external validity. The model population reflects patients with early-stage generalized MG and should not be extrapolated to purely ocular or refractory disease.

There is also uncertainty surrounding key externally derived parameters. Procedure costs for thymectomy were estimated from a single NHS Trust and were based on VATS; thus, generalizability across the UK and to alternative surgical approaches remains uncertain, although national tariff costs and a higher procedure-cost scenario were explored. The mortality risk applied to myasthenic crisis was derived from a US hospitalized cohort and may not fully reflect contemporary UK case mix or service configuration; this parameter was varied in deterministic sensitivity analysis and identified as an influential driver of the results.

Finally, due to a lack of robust data, the base case did not fully incorporate the impact of corticosteroid-related complications. Exploratory analysis suggests this omission likely results in conservative estimates of the cost-effectiveness of thymectomy.

Productivity losses and wider societal costs were excluded in line with the NICE reference case. Given the working-age population affected by MG and the association between disease severity and work impairment, this may also underestimate the broader economic benefits of thymectomy.

Despite these limitations, these findings have important implications. For clinicians and patients, thymectomy is not only clinically effective but also represents an efficient use of health care resources. For policymakers and commissioners, our results support the positioning of thymectomy as an early intervention within the treatment pathway, with strong potential to reduce long-term costs for the NHS while improving patient outcomes and to avoid the potential long-term harms associated with immunosuppressive therapies.

## Conclusions

In this economic evaluation of thymectomy compared with prednisone alone, thymectomy was found to be a highly cost-effective treatment strategy in an NHS setting, offering improvements in quality of life at a lower lifetime cost compared with pharmacological management alone. These findings provide robust economic evidence supporting the routine use of thymectomy in eligible patients with nonthymomatous MG in the UK NHS and establish it as a relevant benchmark against which future therapies should be evaluated within NICE appraisals.

## Notes

Supplement 1. **eFigure 1.** One-Way Sensitivity Analysis—Base-Case (Loss of Response) **eFigure 2.** Threshold Analysis—Treatment Maintenance Period **eTable.** Model Event Analysis (Per Patient)—Base-Case (Loss of Response)

Supplement 2.
Data Sharing Statement
