# Supplementary material for: Economic Evaluation of Thymectomy for the Treatment of Nonthymomatous Myasthenia Gravis
Source: JAMA Netw Open. 2026 Apr 13;9(4):e266612. doi: 10.1001/jamanetworkopen.2026.6612 (PMC13077509; doi:10.1001/jamanetworkopen.2026.6612)
Supplement: Supplement 1. — eFigure 1. One-Way Sensitivity Analysis—Base-Case (Loss of Response) eFigure 2. Threshold Analysis—Treatment Maintenance Period eTable. Model Event Analysis (Per Patient)—Base-Case (Loss of Response) [file jamanetwopen-e266612-s001.pdf]

Supplemental Online Content

Lord J, Walton M, Murphy P, et al. Economic evaluation of thymectomy for the treatment of nonthymomatous myasthenia gravis. *JAMA Netw Open.* 2026;9(4):e266612. doi:10.1001/jamanetworkopen.2026.6612

eFigure 1. One-Way Sensitivity Analysis—Base-Case (Loss of Response)

eFigure 2. Threshold Analysis—Treatment Maintenance Period

eTable. Model Event Analysis (Per Patient)—Base-Case (Loss of Response)

This supplemental material has been provided by the authors to give readers additional information about their work.

© 2026 Lord J et al. *JAMA Network Open*.

eFigure 1. One-Way Sensitivity Analysis—Base-Case (Loss of Response)

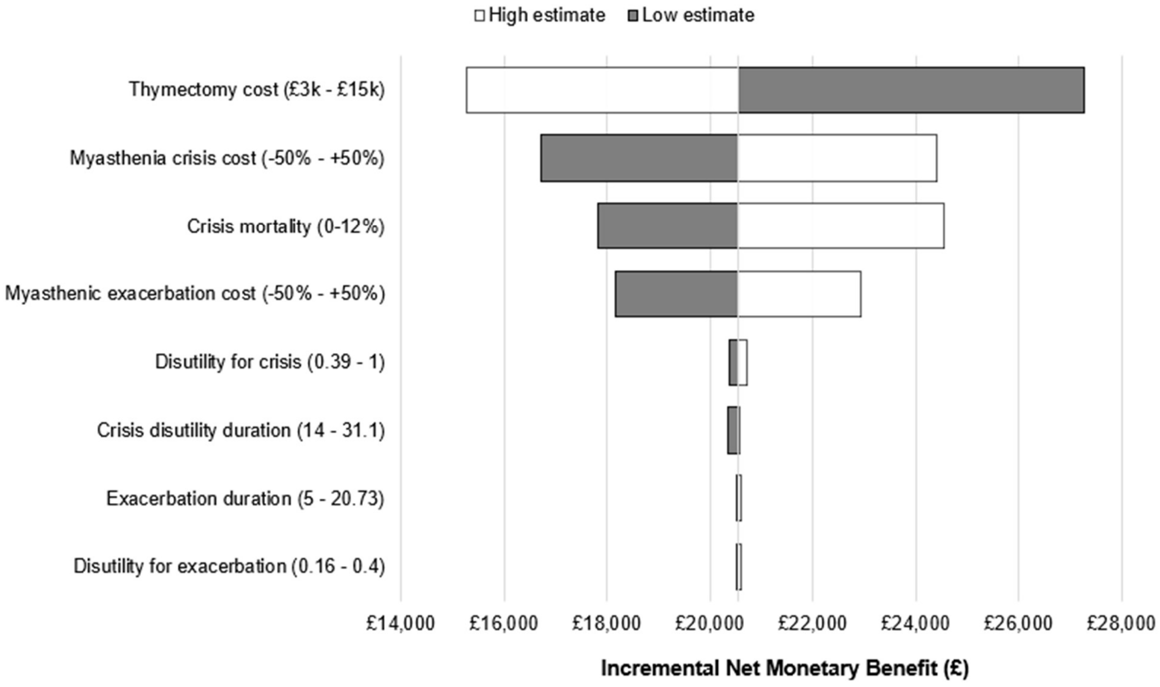

Net monetary benefit based on £25k cost-effectiveness threshold

© 2026 Lord J et al. *JAMA Network Open*.

eFigure 2. Threshold Analysis—Treatment Maintenance Period

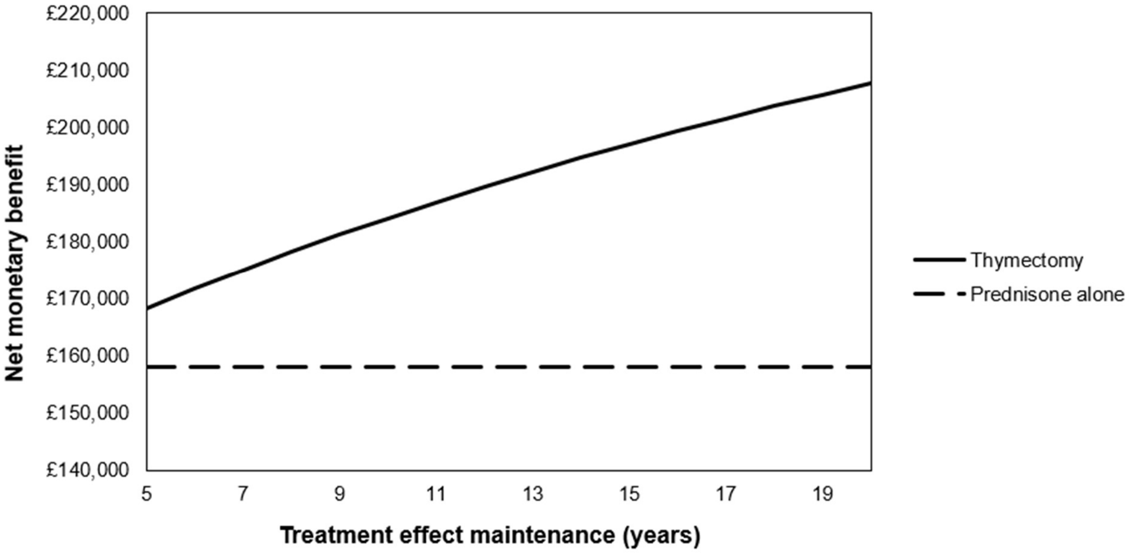

Net monetary benefit based on £25k cost-effectiveness threshold

© 2026 Lord J et al. *JAMA Network Open*.

eTable. Model Event Analysis (Per Patient)—Base-Case (Loss of Response)

	Prednisone alone (PA)	Thymectomy (TPP)	Difference
**Exacerbation events**	11.59	10.91	-0.67
**Crisis events**	3.08	2.78	-0.29
**Average time spent in minimal manifestation (years)**	13.82	16.69	2.87
**Average time spent in mild/moderate (years)**	26.43	24.78	-1.65
**Average time spent in severe (years)**	3.29	2.66	-0.63

© 2026 Lord J et al. *JAMA Network Open*.
