# Supplementary material for: Economic Evaluation of Thymectomy for the Treatment of Nonthymomatous Myasthenia Gravis
Source: JAMA Netw Open. 2026 Apr 13;9(4):e266612. doi: 10.1001/jamanetworkopen.2026.6612 (PMC13077509; doi:10.1001/jamanetworkopen.2026.6612)
Supplement: Supplement 2. — Data Sharing Statement [file jamanetwopen-e266612-s002.pdf]

Data Sharing Statement

Lord. Economic Evaluation of Thymectomy for the Treatment of Nonthymomatous Myasthenia Gravis. *JAMA Netw Open*. Published April 13, 2026. doi:10.1001/jamanetworkopen.2026.6612

Data
Data available: Yes
Data types: Data (not involving human participants)
How to access data: jmlord1@sheffield.ac.uk
When available: With publication

Supporting Documents
Document types: Statistical/analytic code
How to access documents: jmlord1@sheffield.ac.uk
When available: With publication

Additional Information
Who can access the data: While study summary data is reported in the paper, additional requests for data will be considered and provided upon reasonable request.
Types of analyses: Research activities.
Mechanisms of data availability: Following consideration of request.
